# Boosting Performance of Self-Polarized Fully Printed Piezoelectric Nanogenerators via Modulated Strength of Hydrogen Bonding Interactions

**DOI:** 10.3390/nano11081908

**Published:** 2021-07-25

**Authors:** Hai Li, Sooman Lim

**Affiliations:** Department of Flexible and Printable Electronics, LANL-JBNU Engineering Institute, Jeonbuk National University, Jeonju 54896, Korea; lihai@jbnu.ac.kr

**Keywords:** hydrogen bonding interactions, hydrophilic, hydrophobic, β-phase, self-polarized, piezoelectric nanogenerators

## Abstract

Self-polarized piezoelectric devices have attracted significant interest owing to their fabrication processes with low energy consumption. Herein, novel poling-free piezoelectric nanogenerators (PENGs) based on self-polarized polyvinylidene difluoride (PVDF) induced by the incorporation of different surface-modified barium titanate nanoparticles (BTO NPs) were prepared via a fully printing process. To reveal the effect of intermolecular interactions between PVDF and NP surface groups, BTO NPs were modified with hydrophilic polydopamine (PDA) and hydrophobic 1H,1H,2H,2H-perfluorodecyltriethoxysilane (PFDTES) to yield PDA-BTO and PFD-BTO, respectively. This study demonstrates that the stronger hydrogen bonding interactions existed in PFD-BTO/PVDF composite film comparative to the PDA-BTO/PVDF composite film induced the higher β-phase formation (90%), which was evidenced by the XRD, FTIR and DSC results, as well as led to a better dispersion of NPs and improved mechanical properties of composite films. Consequently, PFD-BTO/PVDF-based PENGs without electric poling exhibited a significantly improved output voltage of 5.9 V and power density of 102 μW cm^−3^, which was 1.8 and 2.9 times higher than that of PDA-BTO/PVDF-based PENGs, respectively. This study provides a promising approach for advancing the search for high-performance, self-polarized PENGs in next-generation electric and electronic industries.

## 1. Introduction

With the increase in environmental considerations and demand for next-generation smart wearable electronics, innovative power supply technologies for portable electronics have exhibited considerable potential [1,2,3]. Consequently, numerous attempts have explored innovative energy supply systems [4,5,6], which utilize mechanical motions as novel power sources. One of the energy supply systems is triboelectric nanogenerators, which is triboelectricity that output DC currents [7]. However, piezoelectric nanogenerators (PENGs) [8] have attracted substantial interest due to its high efficiency of converting various forms of abundant irregular mechanical energy into useful electric energy, such as vehicle movements, human body motion, water flow and acoustic waves [8,9,10,11,12]. Furthermore, printing technologies in the fabrication areas of piezoelectric devices are undergoing substantial development due to their ability to be quickly molded for manufacturing structures with any design or shape in a low-cost, single-step and efficient manner [13,14]. To date, an enormous number of studies and continuous progress have attempted to improve the performance of PENGs using various materials [14,15,16].

Among the piezoelectric materials, polyvinylidene difluoride (PVDF) is widely used for fabricating flexible PENGs owing to its high flexibility, easy processing and excellent mechanical properties [17,18,19]. PVDF has the following four distinguishable crystalline phases: a non-piezoelectric α-phase, a strong piezoelectric β-phase, a partially piezoelectric γ-phase and an amorphous δ-phase [20], exhibiting the extraordinary piezoelectric, ferroelectric and pyroelectric properties in electroactive polymers [21,22,23]. In general, the β-phase has the alignment of dipoles perpendicular to the chain axis, resulting in optimum piezoelectric, ferroelectric and pyroelectric properties. [24,25]. For the application of PENGs, to obtain electroactive PVDF films, polarization at a high electric field [26], electrospinning [27], corona poling [28] or mechanical stretching [29] and unfavorable energy consumption processes are required, which are currently the major challenges preventing their widespread applications in energy harvesting.

Recently, various strategies such as the Langmuir–Blodgett deposition method [30], an in situ process [31], phase inversion [32] and the incorporation of fillers [33] have been proposed to achieve high β-phase content in PVDF without applying an external poling process. Of these approaches, the most achievable, scalable and cost-effective mechanism for fabricating PENGs is the incorporation of fillers into PVDF. Therefore, the addition of carbon-related compounds [34], perovskite [35] or inorganic nanoparticles [36] into PVDF to improve the β-phase content has been proposed. In these cases, some of the nucleation mechanisms involved have been explained. Specifically, it was reported that the different surface charge type of the fillers has an impact on the content of β-phase in PVDF. Furthermore, it is also related that the surface charge groups of the nanoparticle could interact with PVDF, resulting in electrical interactions [37]. The use of an appropriate surfactant will lead to the variations in the surface charges on fillers, thereby providing the probability of different content of the β-phase, which can form a hybrid composite film that can completely utilize the β-phase of PVDF and the properties of filler. Moreover, variations in the content of the β-phase caused by hydrogen bonding interactions between the surfactants of nanoparticles and PVDF have also been investigated. Generally, hydrogen bonding interactions are generally formed by hydrophilic tail groups (–OH or –NH_2_) on the nanoparticles reacting with fluorine (F) atoms of PVDF chains [38,39]. However, in our previous finding, we reported that enhanced β-phase content was induced by the reactions between the hydrophobic tail groups (–CF_2_) on the functional BTO NPs and the hydrogen (H) groups of PVDF chains [40]. It is worth noting that our previous finding was based on the condition of the external electric poling process, and the effect of the hydrogen bonding strength was not discussed. To the best of our knowledge, while some efforts have been devoted to improving the performance of self-polarized PENGs by hydrogen bonding interactions, the effect of hydrogen bonding strength on β-phase nucleation has seldom been documented. Therefore, the present work aims to evaluate the influence of the strength of hydrogen bonding on the β-phase formation, enabling a direct correlation between the type of surface group and the nucleation ability of NPs, and thereby creating a new perspective for preparing self-polarized PENGs.

Considering these and our previous findings, BTO NPs with different surface groups were prepared, then were introduced into PVDF matrix to prepare a piezoelectric film. The influence of the BTO NPs surface groups’ type on the mechanical and piezoelectric properties of the composite films was fully explored. For instance, the incorporation of hydrophobic PFD-BTO NPs into PVDF resulted in the highest 90% β-phase content for 8 wt% fillers respected to BTO/PVDF and PDA-BTO/PVDF counterparts due to the stronger hydrogen bonding interactions. In addition, the piezoresponse of the self-polarized PENGs, such as output voltage and power density, is determined mainly by interfacial effects depending on the particle surface group and content. Furthermore, the as-prepared PENG also shows its potential applications by converting mechanical energy into electrical energy.

## 2. Experimental Section

### 2.1. Materials

Dopamine hydrochloride (DAHCl), Tris (hydroxymethyl)aminomethane, polyvinylidene fluoride (PVDF, Mw: 530,000) and 1H, 1H, 2H, 2H-Perfluorodecyltriethoxysilane (PFDTES) were supplied by Sigma-Aldrich (St. Louis, MO, USA). The acetic acid, ethanol and N, N-Dimethylformamide (DMF) used were obtained from Daejung Chemical & Metals Co., Ltd (Siheung, Korea). Barium titanate nanoparticles (BTO, average particle size: 100 nm) were provided by US Research Nanomaterials Inc. (Huston, TX, USA).

### 2.2. Preparation of PDA-BTO Nanoparticles

To prepare surface-modified BTO NPs using hydrophilic PDA, 0.049 g of Tris (hydroxymethyl) aminomethane was dissolved in 40 mL of deionized water. To this, 0.2 g of BTO NPs were then added and ultrasonically treated for 30 min. Subsequently, 0.24 g DAHCl was added into the solution and stirred at 60 °C for 12 h. Thereafter, the resultant PDA-BTO NPs were collected by centrifugation, then ethanol and DI were used to wash them several times. Finally, the PDA-BTO NPs were dried in a vacuum oven (SH-VDO-30NH, SH Scientific, Sejong, Korea) (60 °C for 24 h) to obtain a black powder.

### 2.3. Preparation of PFD-BTO Nanoparticles

To prepare surface-modified BTO NPs using hydrophobic PFTDES, 0.3 g of BTO was added into the 20 mL of ethanol/water solvent mixture (95/5 *v*/*v*). Then, the solution was ultrasonically treated for 30 min. Into the mixture solution, 15 mg of PFDTES and 40 mg of acetic acid were added, respectively. Subsequently, the mixture was ultrasonically treated for 2 h. The PFD-BTO NPs were isolated via centrifugation and ethanol was used to wash them several times. Finally, the PFD-BTO NPs were dried in a vacuum oven (SH-VDO-30NH, SH Scientific, Sejong, Korea) (60 °C for 24 h) to obtain a pale-yellow powder.

### 2.4. Preparation of PENGs

**Figure 1:**
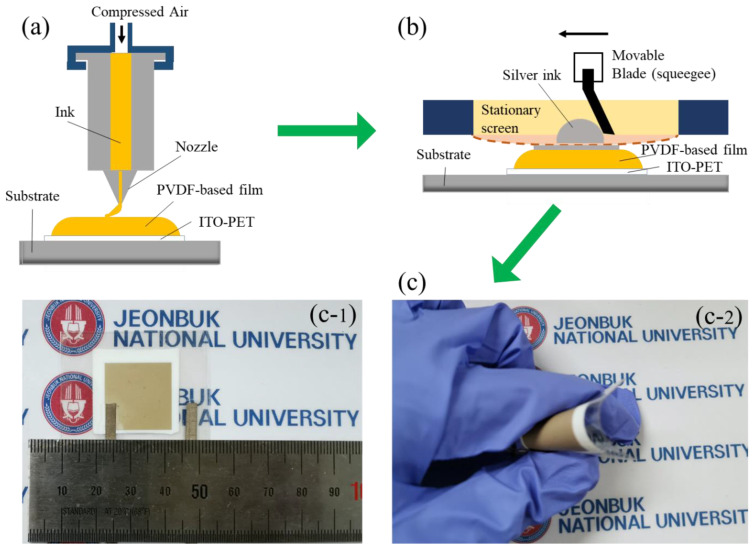
Schematic illustration of (**a**) 3D printing process for PVDF-based film, (**b**) screen-printing process and (**c**) the optical images of the fully printed piezoelectric nanogenerators (**c-1** and **c-2** show actual size of the all-printed device and its flexibility).

To prepare the composite films, 0.08 g of PFD-BTO NPs were dispersed in 10 mL of DMF solution via sonication. Then, 1 g of PVDF was added into the suspensions and stirred at 40 °C for 12 h. The obtained PFD-BTO/PVDF solutions were transferred to a syringe with a 22-gauge nozzle (inner diameter: 0.41 mm) in a dispensing system. The PFD-BTO/PVDF solution was deposited on the ITO-PET film. After printing, the as-printed sample was dried in a vacuum oven (60 °C for 12 h). The size of the as-printed sample was 2.5 × 2.5 cm; the thickness was 30 μm. Subsequently, the top electrode layer of film was printed by a commercial silver paste (SH-VDO-30NH, SH Scientific, Sejong, Korea) using screen printing (area: 2 cm × 2 cm). The full printing process is illustrated in Figure 1a,b. Further, the sandwich-structure device was completely and tightly encapsulated by commercial PET tapes (thickness of 0.08 mm, DUCKSUNG Co., Seoul, Korea) and doctor blade (DUCKSUNG Co., Seoul, Korea) (Figure 1c). The preparation of BTO/PVDF and PDA-BTO/PVDF PENGs used the same preparation method.

### 2.5. Measurement and Characterization

The structure of as-papered samples was investigated using Fourier-transform infrared spectroscopy (Bomen MB 100) with a wavenumber range of 400–4000 cm^−1^. The crystalline structure of the as-papered samples was texted by the X-ray diffraction patterns using Cu Kα radiation (D8 Advance diffractometer, Bruker, Ettlingen, Germany). The morphologies of the as-printed films were performed on a field emission scanning electron microscopy (SUPRA 40VP, Carl Zeiss, Jena, Germany) at the Center for University-Wide Research Facilities (CURF) at Jeonbuk National University. The thermal properties of as-printed films were evaluated by the differential scanning calorimetry (DSC200PC, Netzsch, Germany) under a flowing nitrogen atmosphere, the temperature range is 50–200 °C, and the heating and cooling rates are 10 °C/min. A universal testing machine (Instron 5943, Canton, MA, USA) was used to measure the mechanical properties of as-printed films at a test velocity of 10 mm/min. The specimens were printed into rectangular strips (size: 10 mm × 60 mm, thickness: 50–70 μm). The effective Young’s modulus (E) was obtained by using Hook’s law in the strain between 0 and 1%. A quasi-static d_33_ meter (YE2730A, Sinocera, Yangzhou, China) was used to measure the piezoelectric coefficient of the as-printed films. The output voltages of the printed PENGs were collected using an oscilloscope (KEYSIGHT DSOX2012A, Keysight, Santa Rosa, CA, USA) and the output currents were acquired via a Keithley 2450 source meter (Keithley Instruments, Inc., Cleveland, OH, USA).

## 3. Results and Discussion

**Figure 2:**
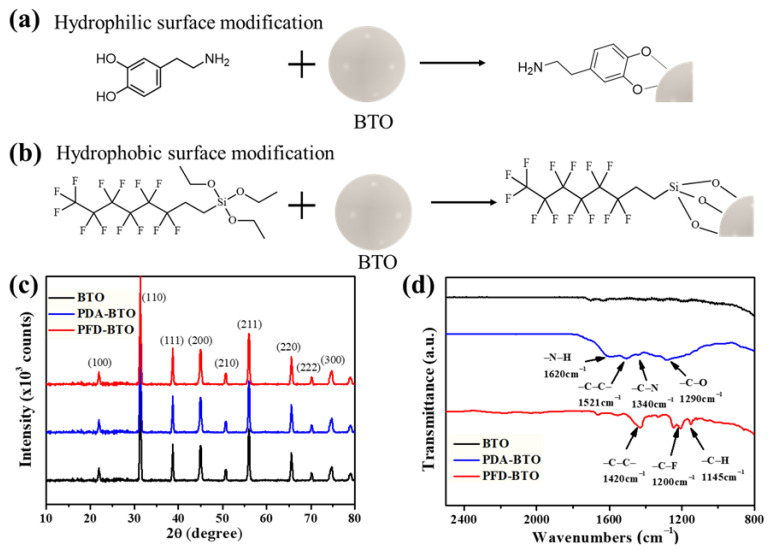
Schematic of the surface (**a**) hydrophilic and (**b**) hydrophobic modifications of BTO NPs. (**c**) XRD and (**d**) FTIR spectra of BTO NPs with surface hydrophilic and hydrophobic modifications.

To investigate the effect of hydrogen bonding interaction on the performance of PENGs, NPs with two different surface functional groups were prepared. Figure 2a shows that the PDA layer was formed on the BTO NPs surface, caused by the strong adhesive capability and self-polymerization of dopamine. Figure 2b depicts the chemical bonding between the PFDTES and BTO NPs, where the PFD-BTO NPs occur by reacting with silanol groups formed during PFDTES hydrolysis. The XRD patterns of the pristine BTO, PDA-BTO and PFD-BTO NPs are shown in Figure 2c. The BTO, PDA-BTO and PFD-BTO NPs exhibited identical diffraction peaks at 22.2, 31.6, 39.1, 45.3, 50.8, 56.2, 65.8, 70.6 and 74.7°, corresponding to the (100), (110), (111), (200), (210), (211), (220), (300) and (300) crystal planes of pure perovskite BTO, respectively, which are well indexed to the JCPDS (#073644) ICDD pattern. The peak splitting at 45° in the XRD pattern is caused by off-centered Ti^4+^ ions, owing to the tetragonal non-centrosymmetric phase in BTO. The results indicated that the BTO, PDA-BTO and PFD-BTO NPs were in the tetragonal phase, and surface modification did not alter the crystal phase of the BTO NPs. Furthermore, FTIR spectra of the BTO, PDA-BTO and PFD-BTO NPs were shown in Figure 2d. After PDA modification, new absorption peaks appeared at 1290, 1340, 1521 and 1620 cm^−1^, which belonged to the C–O, C–N, C–C and N–H bending and stretching vibrations, respectively, indicating the successful surface modification of the PDA-BTO NPs. Moreover, the appearance of the absorption peaks at 1145 cm^−1^ (C–H stretching), 1250 cm^−1^ (C–C stretching) and 1420 cm^−1^ (C–F stretching) signifies the successful surface modification of the PFD-BTO NPs.

**Figure 3:**
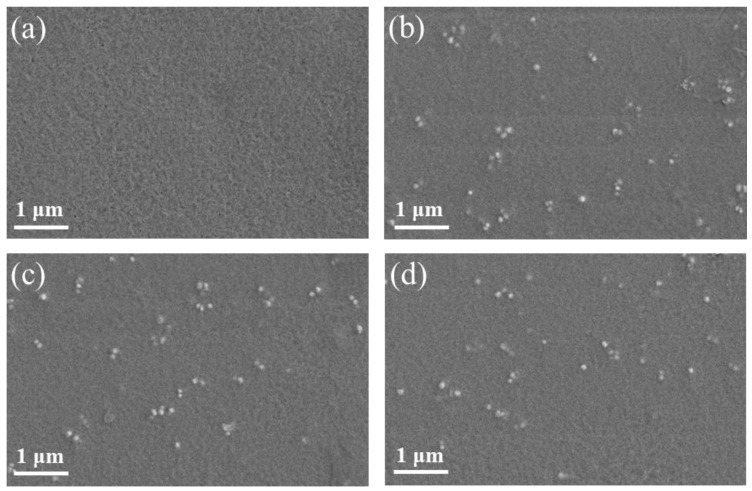
FESEM images of the surfaces of the as-printed films: (**a**) pure PVDF, (**b**) 8 wt% BTO/PVDF, (**c**) 8 wt% PDA-BTO/PVDF and (**d**) 8 wt% PFD-BTO/PVDF.

To eliminate the interference of the agglomeration of high-content NPs that could lead to a decrease in efficient particle–polymer interactions caused the poor performance of composite film [41], 8 wt% NPs were used to prepare the composite film. The dispersion properties of the inorganic NPs in the composite films after surface hydrophilic and hydrophobic modification were investigated, the surface morphologies of the pure PVDF, BTO/PVDF, PDA-BTO/PVDF and PFD-BTO/PVDF composite films were depicted in the FESEM micrographs (Figure 3). Obviously, no visible interface hole defects were detected in any of the samples. The BTO, PDA-BTO and PFD-BTO NPs exhibited good dispersion in the polymer matrix because of the low NP concentration. However, there is a visible difference in the as-prepared composite films when the concentration of the inorganic NPs is high. As shown in Figure S1, there were considerably more aggregated clusters of BTO NPs and PVDF in the BTO/PVDF and PDA-BTO/PVDF composite films than in the PFD-BTO/PVDF composite films when the 20 wt% NPs were used. These aggregations are ascribed to their larger surface areas and higher surface activities. This result indicates that the hydrophobic surface functionalization of inorganic NPs can improve the dispersion of NPs in the composite film more effectively. This phenomenon may be due to the stronger chemical bond between the –CH bond of PVDF and the –CF bond on the PFD-BTO NPs’ surface during mixing, which substantially enhanced the phase compatibility of the composites.

**Figure 4:**
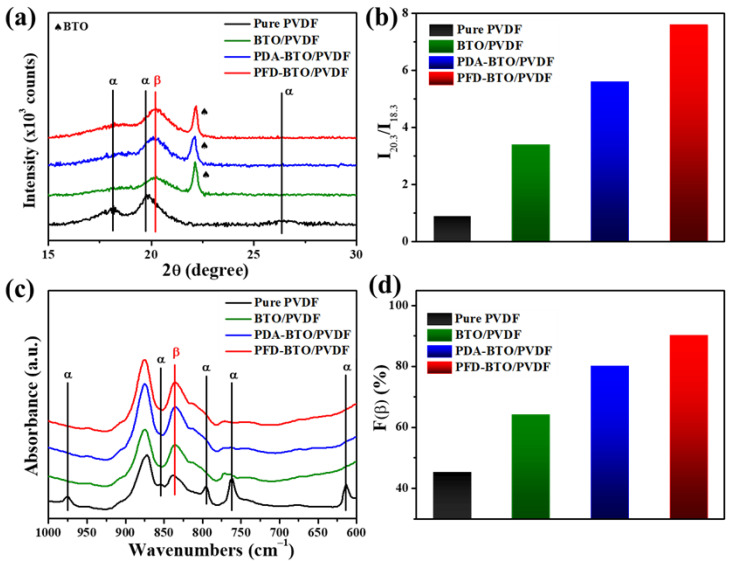
XRD spectra of (**a**) PVDF-based films with 8 wt% filler, (**b**) Ratio of I_20.3_ and I_18.3_ of the as-printed films. (**c**) FTIR spectra of PVDF-based films with 8 wt% filler and (**d**) the F(β) value of as-printed films.

The piezoelectricity of PVDF polymers is determined mainly by the electroactive β phase content of PVDF. To confirm the phase composition changes in the PVDF-based films, the crystalline phase composition of the as-printed films was analyzed using XRD, as shown in Figure 4a. The nonpolar α-phase peaks in the XRD patterns were observed at 18.3° (020), 19.9° (021) and 26.6° (201/310), respectively. Furthermore, the characteristic peak of the polar β-phase appears prominently at 20.3° (110/200), which correlates well with the literature [24], indicating that the pure PVDF film is dominated by the α-phase. However, after incorporating BTO NPs into the PVDF matrix, the intensity of the α-phase peaks at 18.3, 19.9 and 26.6° in the composite films were quenched, regardless of the BTO NPs’ type. Correspondingly, the β-phase of the composite films can be observed via the detection of the (110/200) peak located at 20.3°. This indicates that the inclusion of BTO or PDA-BTO or PFD-BTO NPs into the PVDF matrix could promote the formation of the β-phase in the composite film, indicating that the BTO NPs can act as the nucleating agents of the electroactive β-phase during the PVDF crystallization. Furthermore, we derived a qualitative concept of the β-phase and α-phase contents by calculating the intensity ratio of I_20.3_ and I_18.3_, as shown in Figure 4b. In particular, the types of surface functional groups on the BTO NPs significantly influenced the β-phase content. Unlike the pure PVDF film, which exhibits an intensity ratio (I_20.3_/I_18.3_) of 0.8, the composite films with 8 wt% BTO, PDA-BTO and PFD-BTO NPs exhibit intensity ratios (I_20.3_/I_18.3_) of 3.2, 5.5 and 7.6, respectively. These results confirm that the incorporation of hydrophobic PFD-BTO NPs into the PVDF matrix can lead to a more efficient formation of the β-phase, which may be attributed to stronger hydrogen bonding interactions between the surface agent of PFD-BTO NPs and the –CH_2_ groups of the PVDF chains.

The phase composition of the PVDF-based films was further investigated via FTIR spectroscopy, as shown in Figure 4c. The characteristic peaks at 614, 764, 855 and 976 cm^−1^ correspond to the nonpolar α-phase, while the vibrational peaks at 840 cm^−1^ are ascribed to the polar β-phase [42]. Apparently, the characteristic absorption intensity of the α-phase peak of all the composite films weakened after BTO NPs were added into the PVDF matrix, regardless of the BTO NP types. Notably, the characteristic absorption intensity of the β-phase located at 840 cm^−1^ become more pronounced, manifesting that the inclusion of the BTO or PDA-BTO or PFD-BTO NPs can improve the β crystal phase content in the nanocomposite film. These phenomena were consistent with the XRD results.

In order to quantitatively characterize the polar β-phase content of the PVDF-based nanocomposite film, the relative fraction of the β-phase (F (β)) in all the samples was determined from the absorbance at 764 and 840 cm^−1^ [43,44,45]. The crystalline content of the polar β-phase in the PVDF-based films can be calculated using the following Beer–Lambert law [24]:

(1) $$F\left(\beta \right)=\frac{A_{\beta }}{\left(\frac{K_{\beta }}{K_{\alpha }}\right)A_{\alpha }+A_{\beta }}$$

where A_α_ and A_β_ are the absorbance values at 763 and 840 cm^−1^, respectively. K_β_ (7.7 × 10^4^ cm^2^ mol^−1^) and K_α_ (6.1 × 10^4^ cm^2^ mol^−1^) are the absorption coefficients of the β-phase and α-phase, respectively. The variation in the F(β) values due to the inclusion of different surface functional BTO NPs is shown in Figure 4d. Compared with the pure PVDF film, which has an F(β) value of 45.42%, the nanocomposite films with an 8 wt% BTO NPs content with different surface functional groups exhibit F(β) values of 56.25, 80.38 and 90.21%. These results further confirm that the hydrophobic surface functionalization strategy of nanoparticles can more effectively promote the formation of the β-phase than the hydrophilic surface functionalization counterpart. In addition, the effect of various PFD-BTO NPs contents on the β-phase was also investigated, as shown in Figure S2. As the PFD-BTO NPs content increased from 2 to 8%, the β-phase content of the PFD-BTO/PVDF composite film marginally improved from 82 to 90%. However, a slight decrease in the electroactive β-phase content was observed when the filler (PFD-BTO NPs) content in the polymer matrix increased from 8 to 10 wt%. This reduced β-phase content in the nanocomposite film caused by the over-addition (10 wt%) of the PFD-BTO NPs is mainly attributed to the shielding effect of the NPs [41].

**Figure 5:**
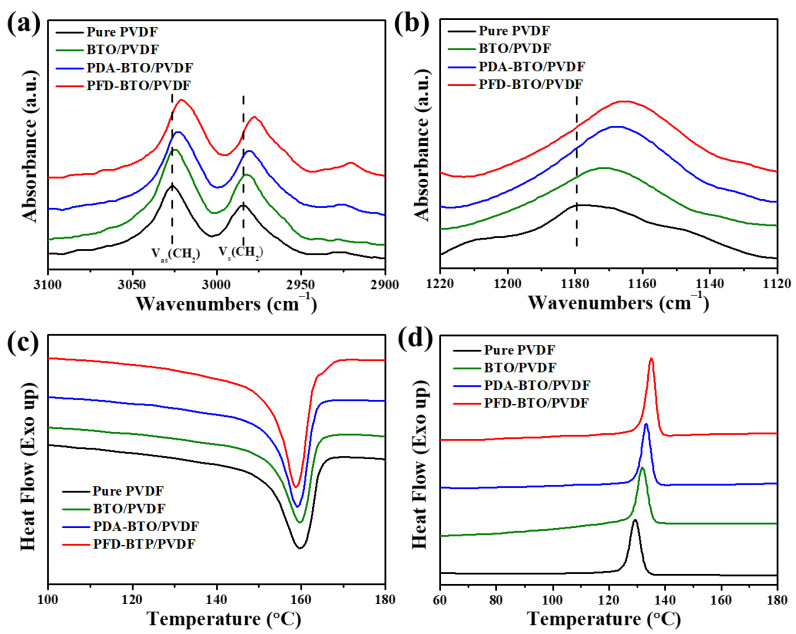
FT-IR spectra of the as-printed pure PVDF, BTO/PVDF, PDA-BTO/PVDF and PFD-BTO/PVDF films with a wavenumber range of (**a**) 3100 to 2900 cm^−1^ and (**b**) 1220 to 1120 cm^−1^. DSC (**c**) melting and (**d**) crystallization curves of the as-printed samples.

Previous studies have demonstrated that the formation of the β-phase can be caused by specific intermolecular interactions, such as ion-dipole and hydrogen bonding interactions. To deepen the understanding of the influence of interfacial interactions by the inclusion of BTO NPs, Figure 5 shows the more detailed information of a shift in the FTIR peak position of the PVDF-based films, which is related to the interactions between the fillers and PVDF. The asymmetric (ν_as_) and symmetric (ν_s_) stretching vibration bands of –CH_2_ in PVDF associated with the wavenumber range from 3100 to 2900 cm^−1^ has a strong sensibility with the hydrogen bonding situation [46,47]. Notably, in the composite, the two fundamental vibrational bands for the ν_as_ (–CH_2_) and ν_s_ (–CH_2_) of the PFD-BTO/PVDF film shifted toward the lower wavenumber side, comparative to the BTO/PVDF and PDA-BTO/PVDF counterparts (Figure 5a), signifying a stronger reduction in the vibrational frequency of the –CH_2_ band, which indicates the stronger hydrogen bonding interactions between the BTO NPs and PVDF. Similarly, a more obvious red shift is detected in the absorption wavenumber at 1180 cm^−1^ of the –CF_2_ stretching after the inclusion of the PFD-BTO NPs, as shown in Figure 5b. These results indicate that the inclusion of the PFD-BTO NPs into PVDF induces strength enhancing of the hydrogen bonding interaction, which leads to the higher β-phase content.

DSC was used as a complement of the other identification techniques to further determine the interface hydrogen bonding effects, as shown in Figure 5c,d. The endothermic peaks (melting temperature, T_m_) of the PVDF-based films after the inclusion of the BTO, PDA-BTO or PFD-BTO NPs were 162.34, 161.37 and 160.93 °C, respectively, which are all lower than that of the as-printed pure PVDF film (163.13 °C), as shown in Figure 5c. Many factors, such as the molecular structure, inclusion of fillers and interfacial interactions, could affect the T_m_ of polymeric crystals [24,48,49]. Nevertheless, the lowering of T_m_ may be attributed to the improvement in the β-phase content because the T_m_ of the nonpolar α-phase is higher than that of the polar β-phase. In this case, the inclusion of surface hydrophobic functional PFD-BTO NPs induces a more obvious decrease in T_m_, which corresponds to the higher β-phase content, further indicating that the hydrophobic groups on the NPs’ surfaces resulted in the more efficient nanoparticles-polymer chain interactions than other groups on NP surfaces. Meanwhile, the PFD-BTO/PVDF film exhibited a more pronounced shift in the crystallization temperature (exothermic peak, T_c_) toward a higher temperature than the other PVDF-based films (Figure 5d). The increased T_c_ of the PVDF-based composite films with the incorporation of nanoparticles may be due to effective heterogeneous nucleation and the formation of hydrogen bonds between PVDF and the BTO NPs. The hydrogen bonding interaction between PVDF and the BTO NPs can inhibit the movement of polymer chain segments and cause a shift in the T_c_ of the composite films to a higher temperature than that of the pure PVDF film. These results further confirm that the hydrophobic surface functionalization strategy of NPs can facilitate the crystallization kinetics of PVDF due to the stronger hydrogen bonding interactions. The total crystallinity (ΔX_c_) of the as-printed PVDF-based films can be calculated using the following equation, assuming that the heat of fusion of 100% crystalline (ΔH_0_) PVDF is 104.7^−1^ [50]:

(2) $$\Delta X_{c}=\frac{\Delta H_{m}}{\left(1-\theta \right)\Delta H_{0}}$$

where ΔH_m_ is the fusion enthalpy measured and θ is the weight percentage of BTO filler. The fusion enthalpy (ΔH_m_), melting temperature (T_m_), crystallization temperature (T_c_) and degree of crystallinity (ΔX_c_) of the as-printed samples, as obtained from equation (2), are presented in Table 1. As shown in Table 1, the inclusion of the BTO, PDA-BTO, or PFD-BTO NPs in the PVDF matrix can influence the degree of crystallinity of the PVDF-based films. Notably, the ΔX_c_ (44.04) of the PFD-BTO/PVDF film exhibited a higher value than that of the other composite films. These phenomena are caused by the stronger hydrogen bonding interaction between PFD-BTO and PVDF, resulting in a heterogeneous nucleation effect, which promotes the crystallization kinetics of PVDF.

Furthermore, to analyze the influence of different surface functional BTO NPs on the mechanical properties, the mechanical stress–strain response of the PVDF-based films was investigated using a tensile test; the results are plotted in Figure S3. The corresponding Young’s modulus (E), tensile strength (σ_break_) and elongation at the break (ε_break_) are presented in Table 2. It was confirmed that the incorporation of the BTO, PDA-BTO, or PFD-BTO NPs in the polymer matrix causes an increased tensile strength, Young’s modulus and elongation at the break because the filler reinforces the material, as demonstrated by other nanofillers [51]. During stretching, the hydrogen bonding interaction between the BTO NPs and the PVDF chain can prevent deformation. Consequently, the 3D network structure in the composite film absorbs more energy before breaking than the pure PVDF film. Meanwhile, Young’s modulus is 1442 MPa for the PFD-BTO/PVDF film, which is ~64% higher than that of the pure PVDF film. Evidently, the stronger hydrogen bonding interaction can significantly improve the mechanical properties of the composite film, which can resist further deformation.

**Figure 6:**
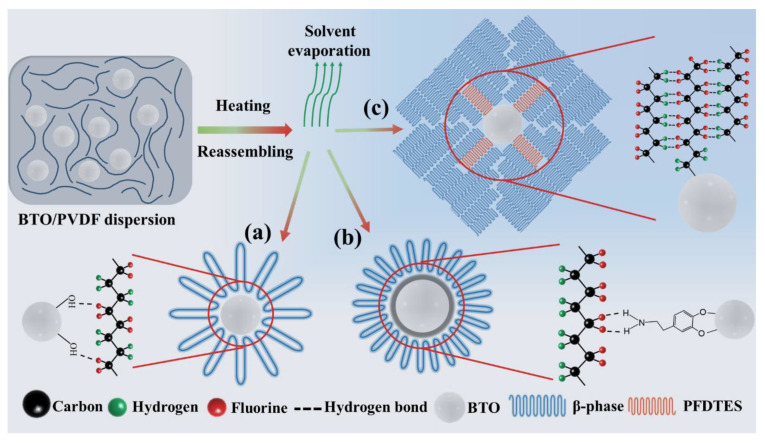
Schematic illustration of the transformation of α-phase to β-phase in the PVDF film by inclusion of (**a**) BTO or (**b**) FDA-BTO or (**c**) PFD-BTO NPs.

Based on the experimental results and the literature review, crystal β-phase nucleation by BTO NPs doping is governed by the hydrogen bonding interactions between the CH_2_/CF_2_ dipoles of the PVDF chains and the surface groups of the NPs; the proposed phase transformation in the PVDF-based films is schematically depicted in Figure 6. Figure 6a schematically depicts the interaction of the −OH group present on the surface of the BTO NPs with F atoms on the PVDF chains. The interaction between these two groups results in the formation of hydrogen bonds, which enhances the β-phase content and crystallinity. Similar behavior was observed in the PDA-BTO/PVDF composite film in which dopamine groups were attached to the BTO NPs, as shown in Figure 6b. During crystallization, the surface of the PDA layer with a large number of amino groups could interact with the –CF_2_ groups in the PVDF chains, forming hydrogen bonding interactions. These hydrogen bonding interactions could induce additional PVDF chain conformation stacking to the β-phase, which restricts the movement of the PVDF chains and increases the activation energy of crystallization [38]. Furthermore, the behavior of these two BTO NPs induced the β-phase in the PVDF matrix to be similar to that in the PFD-BTO/PVDF counterpart. A schematic illustration on the phase transformation (α-phase to β-phase) of the PFD-BTO/PVDF film is depicted in Figure 6c. During the crystallization of the nanocomposite in the solution, dipolar intermolecular interactions exist on the surface hydrophobic groups of the PFD-BTO NPs and PVDF chains between with DMF due to DMF’s high dipole moment (D = 3.82) [52]. The polar moieties of DMF tend to rotate strong dipoles of C–F bonds around the NP surface group chain backbone and the C–C bonds of the PVDF, resulting in an all-trans conformation during the polymer crystallization process and the formation of the electroactive β-phase [53]. The special structure of the surface hydrophobic groups present on the PFD-BTO NPs enable the NPs to induce stronger hydrogen bonding interactions and act as nucleation centers for the β-phase formation in the PVDF matrix. The –CH groups of PVDF reacted with the –CF groups on the surface of the PFD-BTO NPs via the formation of H–F hydrogen bonds when the DMF solvent evaporated. The dipolar intermolecular interactions are replaced by hydrogen bonding interactions to maintain the alignment of the stabilized PVDF chains on the surface of the NPs. After the DMF solvent evaporated, the surface groups present on the PFD-BTO NPs reassembled with the crystal structure of the β-phase in the PVDF, yielding a mortise-tenon joint structure in the nanocomposite film.

**Figure 7:**
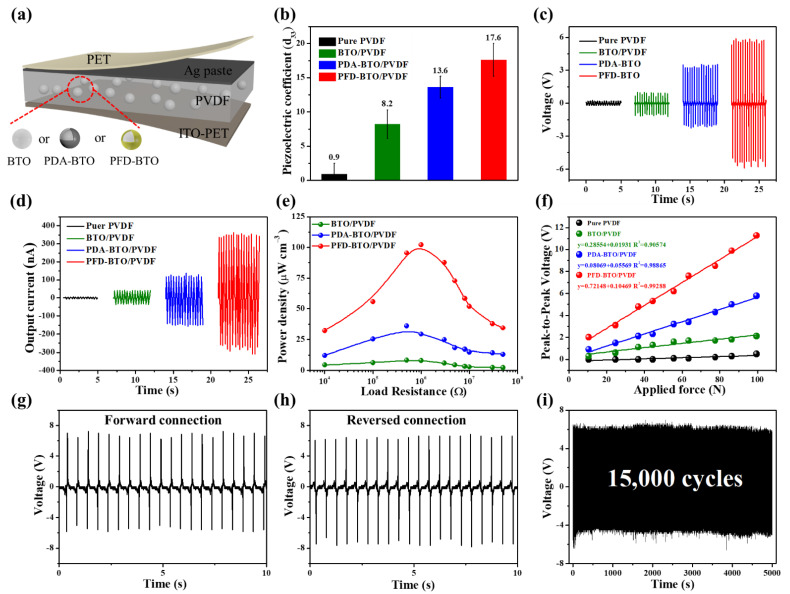
(**a**) Schematic structure of the printed PENGs. (**b**) Piezoelectric coefficient (d_33_) of PVDF-based films with 8 wt% filler content of the BTO, PDA-BTO or PFD-BTO NPs. (**c**) Output voltages and (**d**) output current of all as-printed PENGs under periodic impact force with 100 N at 3 Hz. (**e**) Dependence output power density of all as-printed PENGs on load resistance. (**f**) Dependence of voltage of all as-printed PENGs on the different applied force. Output voltage signals from (**g**) forward-connected and (**h**) reverse-connected circuits. (**i**) Voltage recorded of the PFD-BTO/PVDF-based PENG at 100 N of 3 Hz for 15,000 cycles.

To investigate the piezoelectric properties of the composite films and confirm the applicability and advantages of our proposed approach, the piezoelectric coefficient (d_33_) of the PVDF-based composite films with different surface functional BTO NPs without any requiring poling process was measured using a quasi-static d_33_ meter, as shown in Figure 7b. The results reveal that the incorporation of the BTO, PDA-BTO or PFD-BTO NPs increases the piezoelectric coefficient (d_33_) of the composite films. This enhanced performance correlates with that in previous studies and can be attributed to the enhanced piezoelectric β-phase content because the BTO NPs act as nucleating agents to promote electroactive β-phase formation [37]. In particular, the piezoelectric coefficient is more noticeable for the PVDF films containing surface hydrophobic functional BTO NPs (up to 17.6 pC/N), which is attributed to the higher electroactive β-phase content caused by the stronger hydrogen bonding interactions. Consequently, the incorporation of surface hydrophobic functional PFD-BTO NPs can significantly enhance the piezoelectric properties of the composite films.

To further confirm the superiority of our approach, PVDF-based nanocomposite films with different surface functional BTO NPs were fabricated as PENGs without any poling process. The schematic diagram and optical image of the as-fabricated sandwich-like flexible PENG are shown in Figure 7a, respectively. The assembled PENG, which exhibits excellent flexibility, can be easily crimped by fingers, as shown in Figure 1c. An electromechanical platform was used to further investigate the piezoelectric performance of the printed PENGs and to generate external compressive forces. Under a mechanical compressive force of 100 N and a frequency of 3 Hz, the output voltages and currents of the PENGs during the mechanical compression and release processes are shown in Figure 7c,d. The output voltages of the PVDF-based film after the inclusion of the BTO, PDA-BTO, or PFD-BTO NPs were 0.2, 0.9 and 3.2 V, respectively. However, this enhanced piezopotential is presumably limited by the agglomeration of the increased BTO or PDA-BTO NPs contents in the PVDF matrix and ineffective stress transferability [54]. Furthermore, the output voltage of the PFD-BTO/PVDF-based PENG was 5.9 V (Figure 7c), which is approximately six-fold and twice higher than that of the BTO/PVDF-based PENG and the PDA-BTO/PVDF-based PENG, respectively. Similarly, the output current of the PFD-BTO/PVDF-based PENG exhibited considerably higher values than those of the corresponding BTO/PVDF-based and FDA-BTO/PVDF-based PENGs, as shown in Figure 7d. This higher performance is related to the higher content of the β-phase discussed above. Additionally, the electrical output voltages of the as-prepared PENGs were tested with poling (electric field: 50 kV mm^−1^, 110 °C, 12 h) and without poling under the same mechanical force condition, as displayed in Figure S4. Interestingly, the output voltage of the PFD-BTO/PVDF-based PENG without and with electric poling showed no significant change. However, other PVDF-based PENGs exhibited considerable variations in the output voltages after electric poling. These significant enhancements are due to the strong electric polarization across the composite film of other PVDF-based PENGs. Therefore, it can be revealed that this method of the inclusion of the surface hydrophobic functional PFD-BTO NPs eliminates the demand for conventional electrical poling of the PVDF film to achieve high piezoelectric properties. Moreover, the output voltages of the PFD-BTO/PVDF-based PENG with various PFD-BTO NPs contents at a periodic mechanical impact force (100 N, 3 Hz) and different frequencies (force maintained at 100 N) were measured, as shown in Figure S5. In particular, the 8 wt% PFD-BTO/PVDF-based PENG exhibited a significantly higher output voltage value than the other samples because of its relatively high β-phase content, as shown in Figure S5a. The peak-to-peak value of the output voltage for the PFD-BTO/PVDF-based PENG with 8 wt% PFD-BTO loading is ~11.3 V, which is almost twice that of the PDA-BTO/PVDF-based PENG device (~6.1 V). Therefore, we selected the 8 wt% PFD-BTO/PVDF-based PENG to perform the following piezoelectric test. Moreover, Figure S5b depicts the output voltages of the as-printed PENG at different impacting frequencies under a constant force of 100 N. Evidently, the output voltages of the as-printed PENG increased proportionally with increasing impacting frequencies. This phenomenon is ascribed to the faster strain rate of the piezoelectric film induced by the increasing frequency [55]. To the best of our knowledge, the output performances of the PFD-BTO/PVDF-based PENG are much higher compared to many other self-polarized PENGs, as given in Table S1. Correspondingly, the superior electrical output performance of the PFD-BTO/PVDF-based PENG demonstrates that the incorporation of the surface hydrophobic functional PFD-BTO NPs in the PVDF matrix exhibited a significantly improved electromechanical conversion efficiency.

In addition, to systematically analyze the output power density of the as-prepared PENGs, the output signals were recorded as a function of the external load resistances ranging from 5 kΩ to 50 MΩ. The output power density of the devices can be calculated using P = UI/(RAt), where U, R, A and t represent the output voltage, load resistance, effective area and sample thickness, respectively. As shown in Figure 7e, the output power density of the devices first increased, then decreased with the increase in load resistance. The maximum output power density of the PFD-BTO/PVDF-based PENG reached 102 μW cm^−3^ at a loading resistance of 3 MΩ, which is 10 times and 3 times higher than those of the BTO/PVDF-based PENG and PDA-BTO-based PENG, respectively. Furthermore, Figure 7f depicts the linear relationship between the output voltages and impact force to evaluate the effects of external impact force on the output performance of the PVDF-based composite PENGs. The voltage sensitivity of the as-prepared PENGs can be obtained by the slope of the linear graph using the following equation: S = ∆V/∆F, where ∆V and ∆F are the relative variations in the output voltage signal and the impact force, respectively. The output voltages of all the as-prepared PENGs increased proportionally with an increasing impact force. More specifically, the PFD-BTO/PVDF-based PENGs exhibited higher sensitivity (104.6 mV N^−1^) and voltage linearity (0.99288) than the BTO/PVDF-based PENG and PDA-BTO/PVDF-based PENGs, enabling potential application as a pressure sensor, which indicates that the inclusion of surface hydrophobic functional NPs can significantly improve the sensitivity of PENGs devices.

Further, as a proof-of-concept to verify that the output signals were purely generated by the piezoelectric effect of the as-printed PFD-BTO/PVDF-based PENG, switching-polarity tests were conducted and the results plotted in Figure 7g,h. When the electrode of the as-printed PFD-BTO/PVDF-based PENG was connected to the measurement circuit in the forward direction, a positive electrical signal was generated under the pressure release movement and vice versa. The electrical output signal switching caused by changes in the electrode connection change revealed that the output signals were purely generated by the piezoelectricity of the as-printed PFD-BTO/PVDF-based PENG. Moreover, considering the real-life practical applications, it is necessary to evaluate the stability and durability of the as-printed PFD-BTO/PVDF-based PENG. The mechanical durability of the as-printed PFD-BTO/PVDF-based PENG was verified using a dynamic loading test performed for 15,000 cycles under a pressure of 100 N at 3 Hz, as shown in Figure 7i. The output voltages revealed no fluctuation or attenuation during the repeated loading tests, which could be attributed to the special mortise-tenon joint structure in the PFD-BTO/PVDF composite film. In addition, the values of the output voltage did not exhibit significant changes, even after the as-printed PFD-BTO/PVDF-based PENG was prepared for two months (Figure S6), revealing that the printed piezoelectric sensor exhibited excellent mechanical durability and stability.

**Figure 8:**
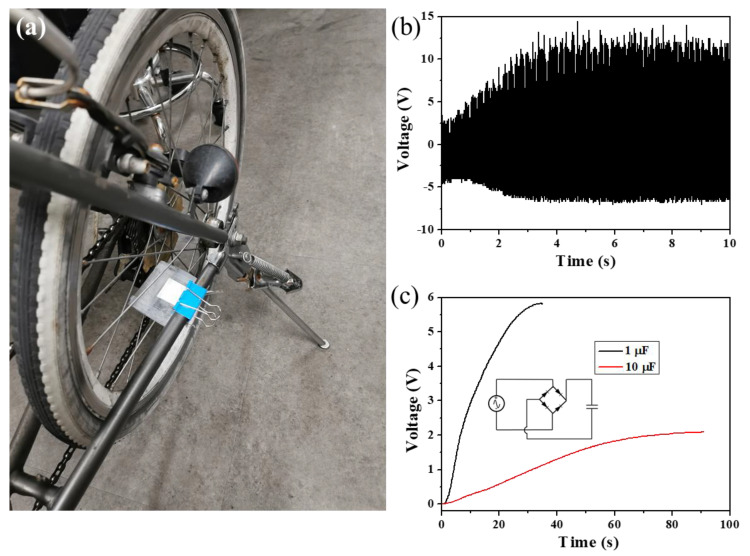
(**a**) PENG placed on a bicycle. (**b**) Output voltage generated from the bicycle. (**c**) Charging voltages of different capacitors; inset: schematic circuit diagram of the commercial capacitor.

Furthermore, to investigate the potential application of the PFD-BTO/PVDF-based PENGs in energy harvesting, the as-papered PENGs were put on the side of a rear wheel and fixed, as shown in Figure 8a. The output voltage was generated by the rotation of the wheel. Furthermore, as the vehicle’s speed increased, the output voltage increased, exhibiting a strong linear relationship, as shown in Figure 8b. The DC outputs with a typical bridge rectifying circuit can be converted by the AC output generated by the as-printed PFD-BTO/PVDF-based PENG, as shown in Figure 8c. The wheel rotation charged the 1 μF and 10 μF capacitors to 5.9 and 2.1 V in 40 s, respectively. Based on these results, the as-printed PFD-BTO/PVDF-based PENG exhibits an optimal power generation performance, revealing that the as-prepared PENGs can be potentially used as a sustainable power source.

## 4. Conclusions

In summary, we have successfully prepared self-polarized PENGs based on PVDF and BTO NPs with different surface groups using a full printing process. The inclusion of surface hydrophobic functional PFD-BTO NPs yielded the highest content of β-phase (90%) for 8 wt% fillers. Furthermore, these results confirmed that the polar β-phase formation is related to the stronger hydrogen bonding interactions between PVDF and the surface groups on the BTO NPs, which also result in a better dispersion of NPs and improved mechanical properties of the composite films. Consequently, the self-polarized PENG prepared from the PFD-BTO/PVDF nanocomposite exhibited a significantly improved electric output than the PVDF, BTO/PVDF and PDA-BTO/PVDF-based PENGs without electric poling. In particular, the maximum output voltage (~5.9 V) and power density (~102 μW cm^−3^) of the PFD-BTO/PVDF-based PENG were 1.8 and 2.9 times that of the PDA-BTO/PVDF-based PENG. In addition, the PFD-BTO/PVDF-based PENG can continue to generate output stably after 15,000 cycles, thus, demonstrating excellent durability. Based on the findings of this study, high-polar-phase-content piezoelectric nanocomposites comprising inorganic nanoparticles and electroactive PVDF, without the need for traditional polarization processes, are novel candidates with superior performance and excellent potential for practical applications.

## Figures and Tables

**Table 1:** Enthalpies of fusion (ΔH_m_), melting temperature (T_m_), crystallization temperature (T_c_) and percentage of crystallinity (ΔX_c_) of PVDF-based films.

	PVDF	BTO/PVDF	PDA-BTO/PVDF	PFD-BTO/PVDF
ΔH_m_ (J g^−1^)	40.72	38.63	41.12	42.42
T_m_ (°C)	163.13	162.34	161.37	160.92
T_c_ (°C)	129.38	131.76	133.19	135.11
ΔX_c_ (%)	38.89	40.11	42.69	44.04

**Table 2:** Mechanical properties of as-printed films. E—Young’s modulus; σ_break_—ultimate tensile strength; ε_break_—strain-to-failure.

	PVDF	BTO/PVDF	PDA-BTO/PVDF	PFD-BTO/PVDF
E (MPa)	878.54 ± 48	1048 ± 70	1257.04 ± 51	1442.28 ± 64
σ_break_ (MPa)	33.46 ± 1.34	34.54 ± 2.65	38.89 ± 3.48	45.83 ± 2.89
ε_break_ (%)	11.39 ± 1.32	11.90 ± 2.19	12.88 ± 1.95	15.33 ± 2.43

## Data Availability

The data are available upon request from the corresponding authors.

## Supplementary Materials

The following are available online at www.mdpi.com/article/10.3390/nano11081908/s1, Figure S1: SEM images of the surfaces of the as-printed films: (a) 20 wt% BTO/PVDF, (b) 20 wt% PDA-BTO/PVDF and (c) 20 wt% PFD-BTO/PVDF. Figure S2: (a) FT-IR spectra of the printed PVDF films with various PFD-BTO nanoparticle contents, (b) The F(β) value variation in the printed samples as a function of the or PFD-BTO nanoparticle content and (c) XRD pattern of the printed PVDF films with various PFD-BTO nanoparticles contents. Figure S3: Stress–strain curves of the pure PVDF, BTO/PVDF, PDA-BTO/PVDF and PFD-BTO/PVDF films. Figure S4: Output voltage output of the pure PVDF, BTO/PVDF, PDA-BTO/PVDF and PFD-BTO/PVDF based PENGs without (a) and with (b) electric poling. Figure S5: (a)Output Voltages of the printed PVDF films with different contents of PFD-BTO nanoparticles with a frequency of 3 Hz and a compress force of 100 N and (b) Output voltages of the 8 wt% PFD-BTO/PVDF based PENGs at different impacting frequencies under a constant pressure force of 100 N. Figure S6: Output voltages of the PFD-BTO/PVDF based PENGs after one month under repeated impact force conditions for 4500 cycles with a frequency of 3 Hz and a pressure of 500 kPa. Table S1: Summary of PVDF-based materials in our study and other reported studies.
