# Supplementary material for: Boosting Performance of Self-Polarized Fully Printed Piezoelectric Nanogenerators via Modulated Strength of Hydrogen Bonding Interactions
Source: Nanomaterials (Basel). 2021 Jul 25;11(8):1908. doi: 10.3390/nano11081908 (PMC8401582; doi:10.3390/nano11081908)
Supplement: Supplementary file 1 [file nanomaterials-11-01908-s001.zip › nanomaterials-1285921-supplementary.pdf]

Supplementary Information

Boosting Performance of Self-Polarized Fully Printed Piezoelectric Nanogenerators via Modulated Strength of Hydrogen Bonding Interactions

Hai Li and Sooman Lim *

Department of Flexible and Printable Electronics, LANL-JBNU Engineering Institute, Jeonbuk National University, Jeonju 54896, Korea; lihai@jbnu.ac.kr

* Correspondence: smlim@jbnu.ac.kr

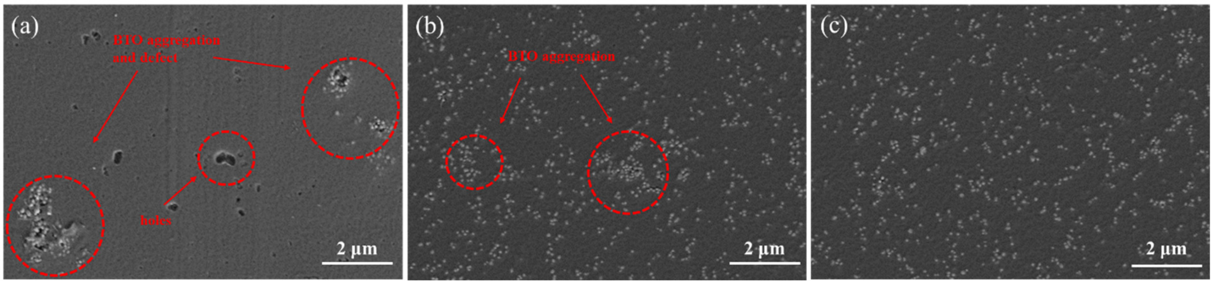

Figure S1. SEM images of the surfaces of the as-printed films: (**a**) 20 wt% BTO/PVDF, (**b**) 20 wt% PDA-BTO/PVDF and (**c**) 20 wt% PFD-BTO/PVDF.

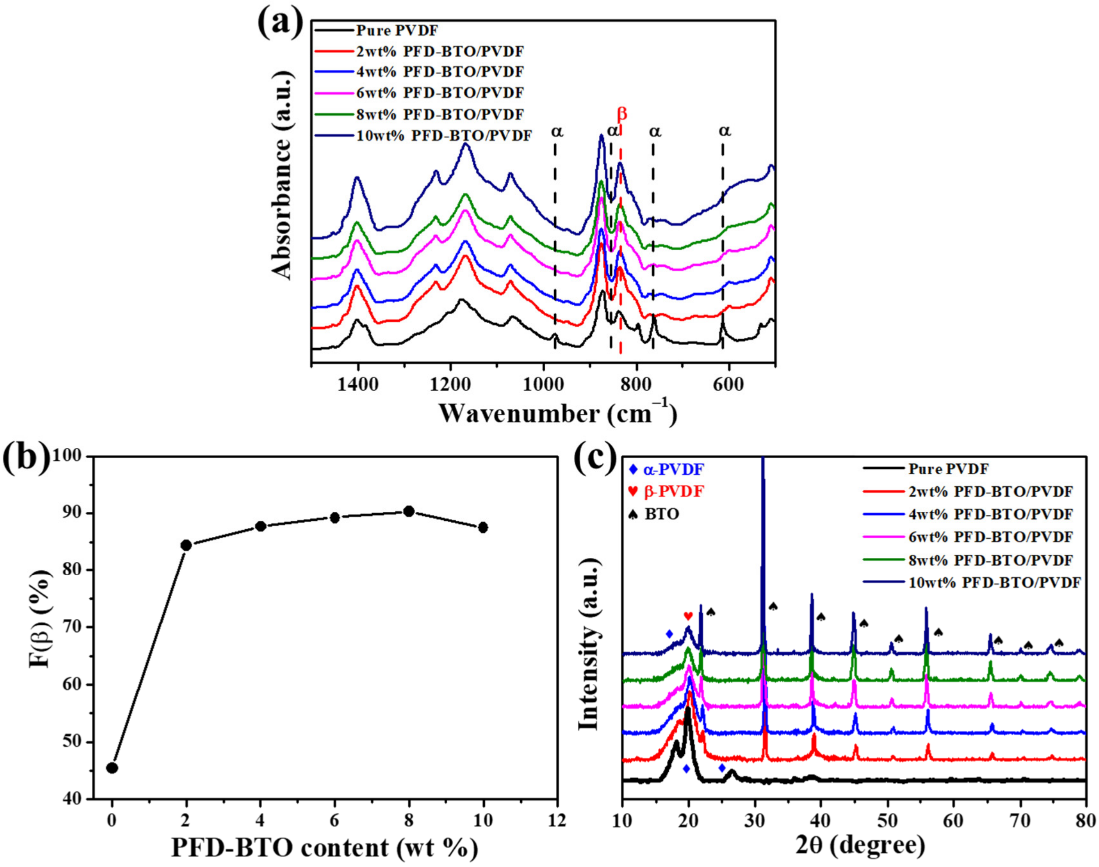

Figure S2. (**a**) FT-IR spectra of the printed PVDF films with various PFD-BTO nanoparticle contents, (**b**) The F(β) value variation in the printed samples as a function of the or PFD-BTO nanoparticle content and (**c**) XRD pattern of the printed PVDF films with various PFD-BTO nanoparticles contents.

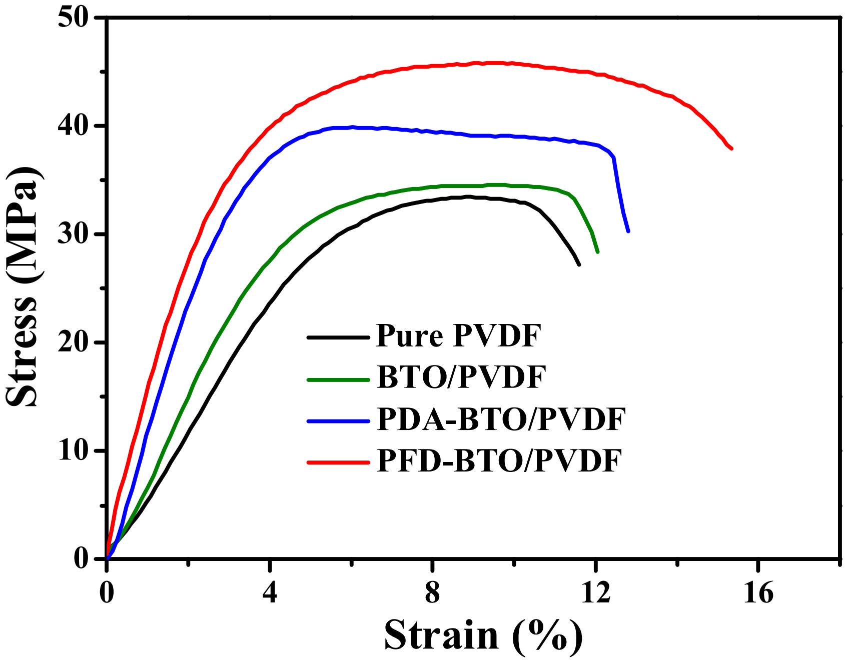

Figure S3. Stress–strain curves of the pure PVDF, BTO/PVDF, PDA-BTO/PVDF and PFD-BTO/PVDF films.

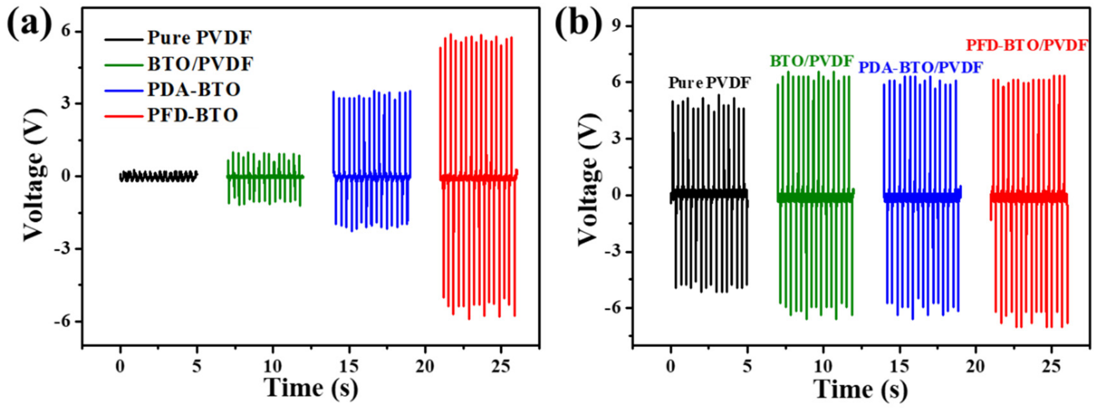

Figure S4. Output voltage output of the pure PVDF, BTO/PVDF, PDA-BTO/PVDF and PFD-BTO/PVDF based PENGs without (**a**) and with (**b**) electric poling

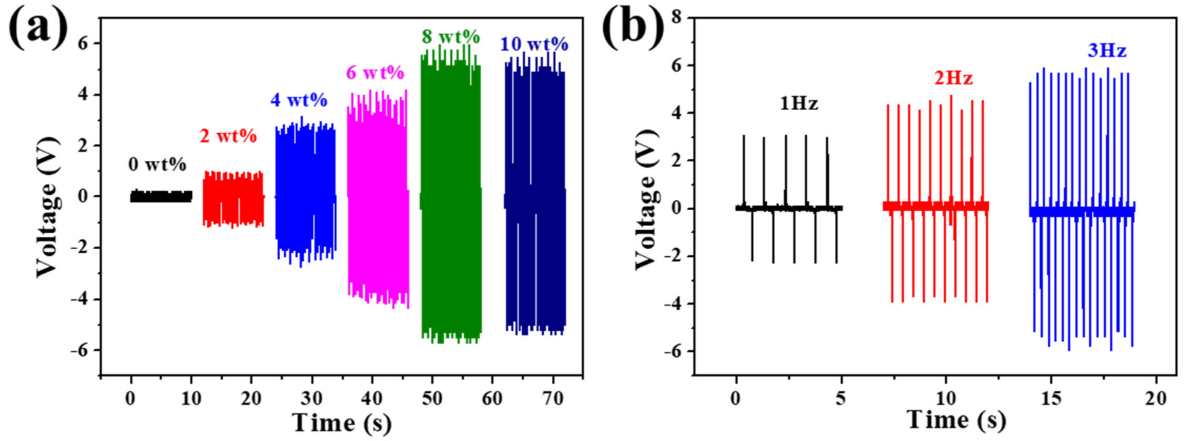

Figure S5. (**a**)Output Voltages of the printed PVDF films with different contents of PFD-BTO nanoparticles with a frequency of 3 Hz and a compress force of 100 N and (**b**) Output voltages of the 8 wt% PFD-BTO/PVDF based PENGs at different impacting frequencies under a constant pressure force of 100 N.

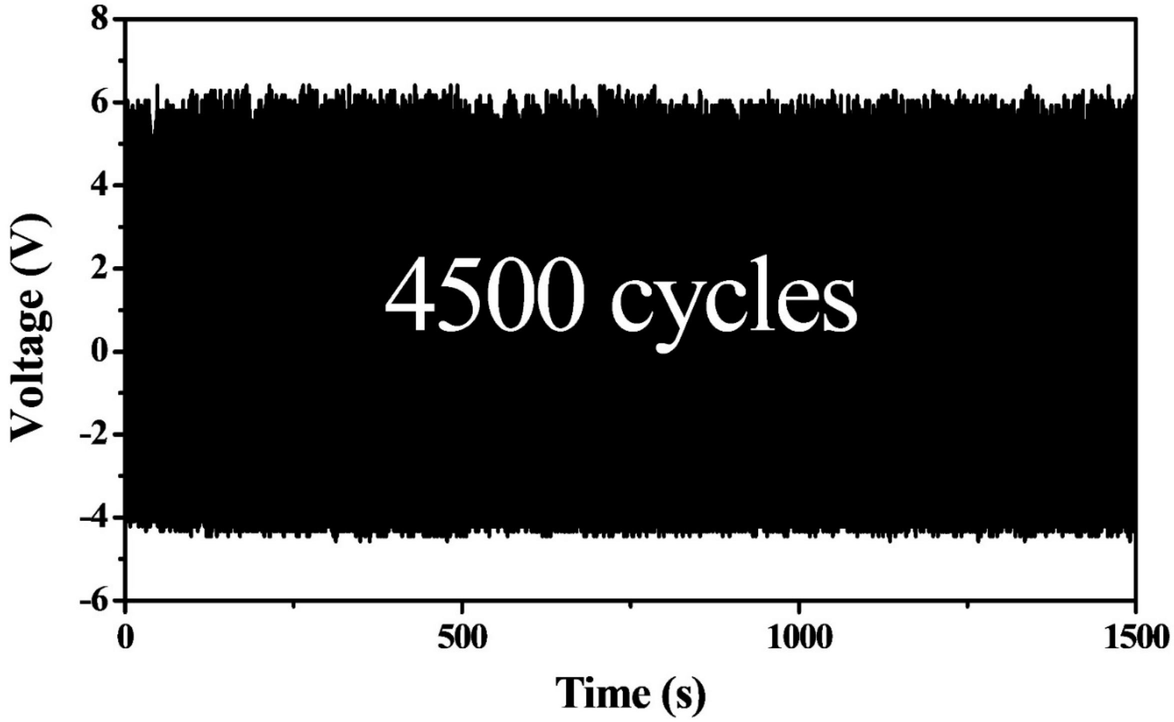

Figure S6. Output voltages of the PFD-BTO/PVDF based PENGs after one month under repeated impact force conditions for 4500 cycles with a frequency of 3 Hz and a pressure of 500 kPa.

Table S1. Summary of PVDF-based materials in our study and other reported studies.

Material	Poling process	Output voltage	Ref.
PDA@BTO/PVDF-TrFE	Yes	6V	1
BTO/PVDF	Yes	6V	2
GAg/PVDF	None	0.1V	3
SWCNTs/PVDF-TrFE	None	1.2V	4
Fe-RGO/PVDF	None	5.1V	5
RGO/PVDF-TrFE	None	2.4V	6
dabcoHReO$_4$/PVDF	None	3.8V	7
PFD-BTO/PVDF	None	5.9V	This work

References

[1]. Guan, X.; Xu, B.; Gong, J. Hierarchically architected polydopamine modified BaTiO3@ P (VDF-TrFE) nanocomposite fiber mats for flexible piezoelectric nanogenerators and self-powered sensors. *Nano Energy*, **2020,** *70,* 104516.

[2]. Zhou, X.; Parida, K.; Halevi, O.; Liu, Y.; Xiong, J.; Magdassi, S.; Lee, P.S. All 3D-printed stretchable piezoelectric nanogenerator with non-protruding kirigami structure. *Nano Energy*, **2020,** *72,* 104676.

[3]. Sinha, T.K.; Ghosh, S.K.; Maiti, R.; Jana, S.; Adhikari, B.; Mandal, D.; Ray, S.K. Graphene-silver-induced self-polarized PVDF-based flexible plasmonic nanogenerator toward the realization for new class of self powered optical sensor. *ACS applied materials & interfaces*, **2016,** *8(24),* 14986–14993.

[4]. Shepelin, N.A.; Sherrell, P.C.; Goudeli, E.; Skountzos, E.N.; Lussini, V.C.; Dicinoski, G.W.; Ellis, A.V. Printed recyclable and self-poled polymer piezoelectric generators through single-walled carbon nanotube templating. *Energy & Environmental Science,* **2020,** *13(3),* 868–883.

[5]. Karan, S.K.; Mandal, D.; Khatua, B.B. Self-powered flexible Fe-doped RGO/PVDF nanocomposite: an excellent material for a piezoelectric energy harvester. *Nanoscale,* **2015,** 7*(24)*, 10655–10666.

[6]. Habibur, R.M.; Yaqoob, U.; Muhammad, S.; Uddin, A.I. Kim, H.C. The effect of RGO on dielectric and energy harvesting properties of P (VDF-TrFE) matrix by optimizing electroactive β phase without traditional polling process. *Materials Chemistry and Physics,* **2018,** *215,* 46–55.

[7]. Li, Q.; Ke, W.; Chang, T.; Hu, Z. A molecular ferroelectrics induced electroactive β-phase in solution processed PVDF films for flexible piezoelectric sensors. *Journal of Materials Chemistry C,* **2019**, *7(6),* 1532–1543.
